# Exploring solutions to improve antenatal care in resource-limited settings: an expert consultation

**DOI:** 10.1186/s12884-022-04778-w

**Published:** 2022-05-30

**Authors:** Carlotta Gamberini, Federica Angeli, Elena Ambrosino

**Affiliations:** 1 Institute for Public Health Genomics, Department of Genetics and Cell Biology, School for Oncology and Developmental Biology (GROW), Faculty of Health, Medicine and Life Sciences, University of Maastricht, Maastricht, The Netherlands; 2 University of York Management School, University of York, York, UK

**Keywords:** Antenatal care (ANC), Expert consultation, Social ecological model (SEM), Maternal and neonatal health (MNH)

## Abstract

**Background:**

Shortage or low-quality antenatal care is a complex and “wicked” problem relying heavily on contextual, socio-cultural, environmental and intersectional aspects. We report the outcome of an expert consultation discussing solutions to improve antenatal care quality, access and delivery in low- and middle-income countries, and providing recommendations for implementation.

**Methods:**

The social ecological model was used as an analytical lens to map and interpret discussion points and proposed solutions. In addition, a conceptual framework for maternal and neonatal health innovation based on the building blocks of the World Health Organization health system and the Tanahashi Health Systems Performance Model provided a logical overview of discussed solutions.

**Results:**

Many barriers and norms continue to hinder antenatal care access. From values, beliefs, traditions, customs and norms, to poor resource allocation, there is a need of reshaping health systems in order to provide high quality, respectful maternal and childcare. The burden of poor maternal health, morbidity and mortality is concentrated among populations who are vulnerable due to gender and other types of discrimination, have financial constraints and are affected by humanitarian crises.

**Conclusions:**

In order to address maternal health issues, good quality and evidence-based services should be guaranteed. Investments in strengthening health systems, including data and surveillance systems and skilled health workforce, should be considered an essential step towards improving maternal health services.

## Background

Pregnancy is a crucial period and the changes happening in this phase have the potential to impact maternal and newborn health immediately, as well as later in life. As such, high quality care during pregnancy (antenatal care, ANC) is important for the health of the mother and the development of the unborn baby. Inadequate care during this time breaks a critical link in the continuum of care, and affects both women and babies [1, 2].

Lack of availability of ANC is a complex, “wicked” problem, which is highly dependent on contextual, socio-cultural, socio-ecological, intersectional aspects, with no straightforward definition or solution [3]. The provision and utilization of ANC services is subject to many different elements, such as lack of quality care; socio-demographic and socio-cultural characteristics of patients, as education, occupation, ethnicity, social relationships and patients’ income level; logistical aspects, such as waiting time and location of facilities; and lastly social perception of general health, illness and diseases [4].

Despite the ambition to reduce global maternal mortality ratio (MMR) to less than 70 per 100,000 live births set by the United Nation Sustainable Development Goal (SDG) [2], the world will fall short of this target by more than 1 million lives with the current pace of progress [5]. The high number of maternal deaths in some areas of the world reflects inequalities in access to quality health services and highlights the gap between high-income countries and low-middle income countries (LMICs), where the burden of MMR is the highest. There is a continued urgent need for maternal health to remain high on the global health and development agenda [6].

To address these issues, experts from different fields working in maternal, newborn and reproductive health gathered for an expert consultation entitled “New approaches to improve antenatal care in resource limited settings”. The event was held virtually on June 14 2021 and was attended by 24 participants from eight countries (United Kingdom, Netherlands, India, Bangladesh, South Africa, Kenya, Ethiopia and Colombia).

The goals were to bring together an interdisciplinary group of experts to discuss solutions to improve ANC quality, access and delivery in LMICs and generally in resource-constrained settings, and to give recommendations for implementation. The first objective was to exchange information and experiences related to ANC across settings, with the use of expert’s presentations. Following the presentations, participants were asked to further discuss in smaller groups the second objective, which was to recognize challenges and experiences of ANC access and delivery in the settings where the experts have worked and practiced in, taking into consideration the different factors that influence health, such those in the social ecological model (SEM) [7]. The third and last objective of the event was to reflect on solutions to improve ANC access and delivery, in a specific setting, or as a general model.

**Fig. 1:**
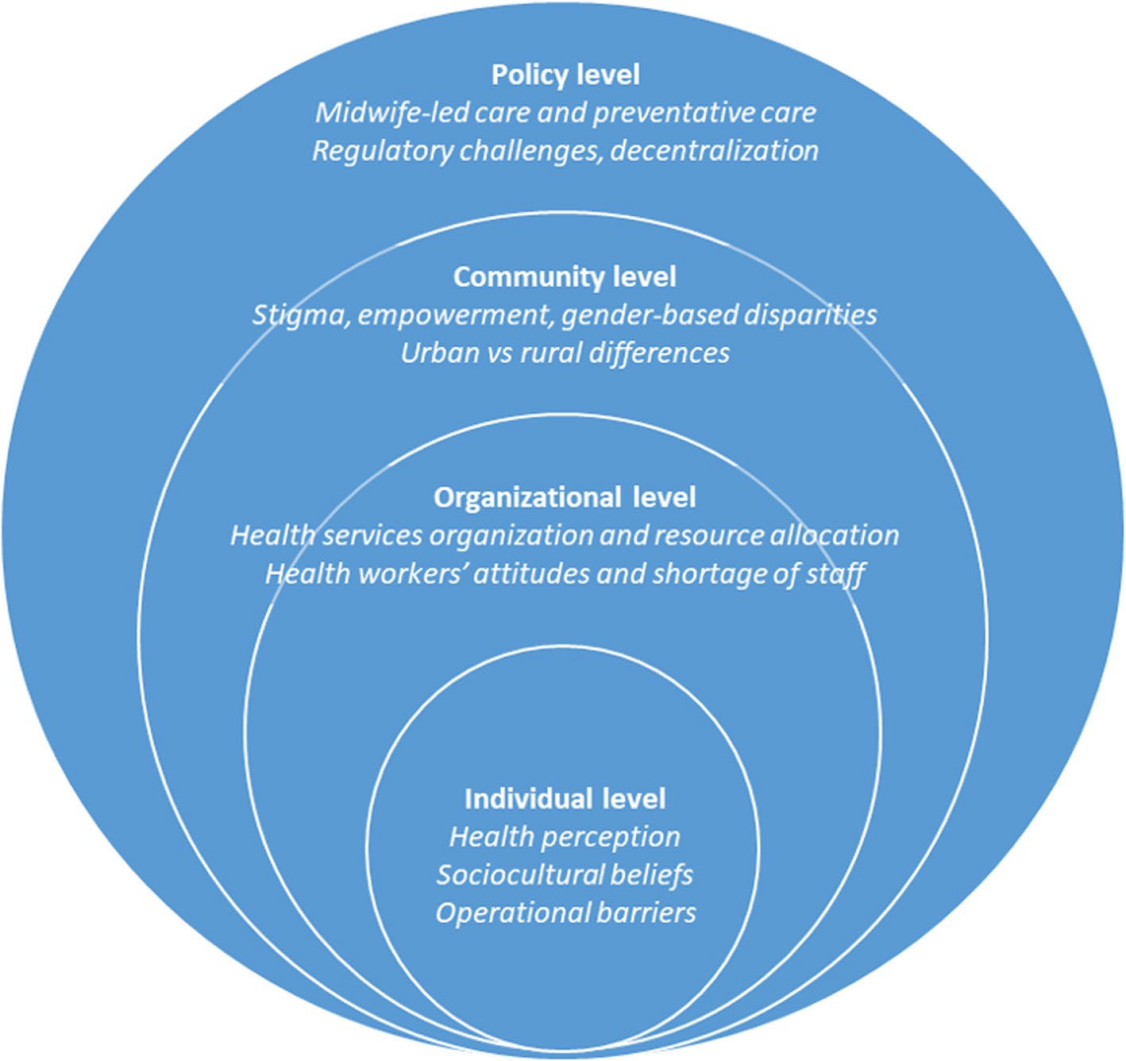
Revised Social Ecological Model, depicting the items discussed in the workshop

**Fig. 2:**
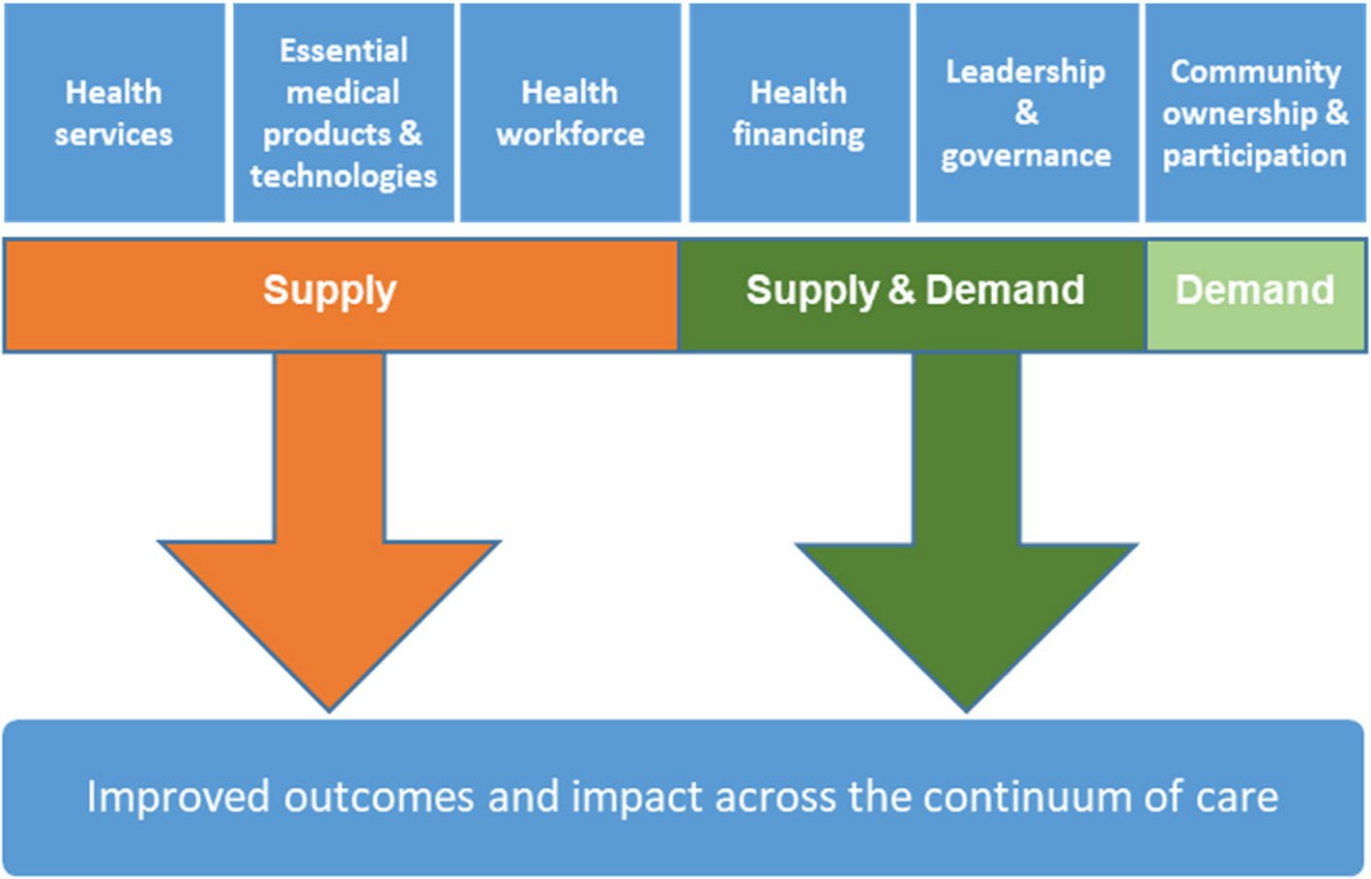
Conceptual framework for Maternal and Newborn health (MNH) innovation adapted from the Tanashi [8]

In order to achieve the second goal, the SEM, shown in Fig. 1, was used as the analytic lens to further discuss the subject, and to organize the study findings. The SEM has five levels: the individual level, which focuses on the person’s attitude and beliefs towards care; the organizational level, which focuses on the role of healthcare systems; the community level, which focuses on the various organizations in the area; and lastly the policy level, which focuses on programs and policies [7]. Finally, a conceptual framework for MNH innovation based on the World Health Organization’s (WHO) health system building blocks and the Tanahashi model of measuring health systems performance (Fig. 2) was chosen to provide a logical overview of the innovations discussed, for the third and last objective [8].

## Methods

### Participants

The 24 participants in attendance at the workshop included experts on maternal, neonatal and child health, sexually transmitted diseases, gynaecology, public health, health systems and services, economics and health technology assessment (Table 1).

**Table 1:** Participants’ affiliations and focus of work

Sector	Country	Field of work
Academic institution	Bangladesh	Maternal, sexual and reproductive health and rights
Hospital institution	Curacao	Obstetrics and gynaecology
Academic institution	Ethiopia	Reproductive, and health services management
Academic institution	India	Gynaecology
Academic institution	Kenya	Public health
Academic institution and non-governmental organization	Netherlands/Kenya	Public health
Academic institution	Netherlands	Health Sciences
Academic institution	Netherlands	Reproductive and maternal health, sexually-transmitted infections
Academic institution	Netherlands	Maternal health and sexually transmitted infections
Academic institution	Netherlands	Health Services Research
Academic institution	Netherlands	Patient care optimization
Academic institution	Netherlands	Sexually transmitted infections
Hospital institution	Netherlands	Gynaecology
Private sector	Netherlands	Design for sustainability
Academic institution	South Africa	Sexual and reproductive health
Academic institution	South Africa	Public health aspects of sexually transmitted infections
Academic institution	South Africa	Respectful maternal care, barriers to quality of care
Academic institution	United Kingdom	Sexual and reproductive health, family planning, HIV/AIDS
Academic institution	United Kingdom	Global health
Academic institution	United Kingdom	Economic evaluation and health technology assessment
Academic institution	United Kingdom	Public relations and management
Academic institution	United Kingdom	Global health
Academic institution	United Kingdom	Public health
Academic institution	United Kingdom	Healthcare management

### Data collection and analysis

Data was extracted from the experts’ presentations, discussing ANC delivery in Europe, South-east Asia, South America and Sub-Saharan Africa. Moreover, additional data was gathered during the second part of the event, when the participants were asked to further discuss the second objective of the workshop in smaller groups.

The audio component of each presentation was recorded using Zoom recording service, with each recording uploaded and stored on a secure university server. Each presentation was transcribed using word processing software (Otter.ai software), and anonymized. No recording was made during the discussions in smaller groups, notes from these discussions were taken by selected participants.

Each transcript and set of notes were subsequently uploaded into Atlas.ti (Version 9.1.3.0, Berlin, Germany). The transcripts and notes served as the basis for the thematic content analysis. The thematic analysis focused on patterns and themes that highlight the subjective viewpoints of the participants. The transcripts were coded using an ‘abductive approach’. Following the initial coding process, codes were grouped into categories to form a working analytical framework, which was used to help code further transcripts. Finally, the SEM and the modified conceptual framework for MNH innovation were utilized to further discuss the subject, and to organize the study findings.

## Results

### Experiences from ANC in different settings

The experiences of ANC in different settings around the globe (Sub Saharan African countries, Eastern Europe, Bangladesh and Colombia) were shared and discussed by five experts in short presentations.

Recurring points conveyed by several speakers were the many barriers that communities encounter in the context of ANC. Although country-level differences were observed, women and their families face similar challenges across settings. The most recognized ones are barriers in access to care, such as those experienced by women living in rural areas experiencing long distances to the healthcare facilities, lack of means of transportation, effects of floods and poor roads.

Another category of barriers that many experts recognized refers to cultural barriers, which were differing the most across settings. Examples of these are lack of awareness or different perceptions of ill health, lack of knowledge about pregnancy complications and ANC, religious practices and beliefs shaping women’s healthcare seeking behaviour, and spousal support and involvement.

Financial barriers were also highlighted as important obstacles that women and their families have to consider. Out-of-pocket payments (direct payments for medical expenses paid by individual users), informal payments and, broadly, poverty and inequality have a substantial effect on the health of mothers and children.

Violence has also a central role in the access to ANC. Besides being a key driver to pregnancy complications, violence (including gender based-violence) leaves mothers with traumatic and stressful experiences often remaining unaddressed.

What transpired from the speakers’ experiences was that a traditional ANC based model, which generally focuses on the clinical assessments and services [9], is becoming inadequate in most LMICs. This observation supports the need for a more tailored ANC to better fit the reality of resource-limited settings, re-evaluating the traditional western model, which has mostly been adopted so far. In most Western countries, ANC has traditionally consisted of a series of one-on-one appointments with a midwife, an obstetrician, or a general practitioner (GP) in a hospital setting. In order to provide ANC based on the individual needs of women, health care providers need to have sufficient resources, and this, in turn, is dependent on organizational norms and values of the setting. Thus, this model is inadequate in LMICs and in general in resource limited settings. All around, experts acknowledged that in order to make the required improvements, healthcare systems should be strengthened, since facilities are often overcrowded, overloaded, they lack skilled healthcare workers, equipment or essential drugs. Further, a need to improve the referral system was pointed out, as well as to strengthen the healthcare service delivery at the point-of-care in order to reach rural areas, and the most vulnerable women.

### ANC access and delivery: challenges and experiences at different levels

#### Individual level

##### Health perception

Across regions, women were reported to often have a limited understanding on the purpose of early ANC and therefore the right time to seek care during pregnancy. This lack of understanding might be influenced by a perception that ANC is primarily provided to detect or treat diseases and was suggested to associate with women’s educational level literacy rate.

Moreover, as the COVID-19 pandemic transformed the way people access health care, with care quickly moving to telehealth and fear of contracting the virus, many women have gone without health care services. Among the discussed settings, this has been particularly the case in India and Bangladesh, where the pandemic has highlighted and aggravated long-standing inequities in healthcare availability and access.

##### Sociocultural beliefs

Individual women’s ANC seeking behaviour was also reported to be restricted by the significant role of family members in health care decisions. Usually, mothers-in-law and husbands are the main decision-makers in determining the need for women’s ANC.

Other important factors influencing the utilization of maternal health services are religious and socio-cultural norms as well as gender stereotypes, which affect the decision-making process of seeking care during pregnancy and postnatal care. The need to attend to domestic chores and care for children at home prevent some women from seeking maternal healthcare services.

##### Operational barriers

Physical barriers, such as living in rural and/or remote areas distant to the health facilities, lack of means of transportation, and obstacles, such as floods and poor roads, are few of the barriers that the experts have identified.

Financial barriers are also important obstacles that women have to consider and might make expectant mothers financially dependent on their families. Indeed, often women are economically reliant on their husbands and are not engaged in any vocation, making it hard to have independency from their families.

From out-of-pocket payments and informal payments, poverty and inequality all have a substantial effect on the health of mothers and children.

#### Organizational level

##### Organization of health services & resource allocation

Poor allocation and shortage of healthcare resources, such as essential drugs and essential equipment, are cited by the experts as one of the challenges to ANC utilization. Additionally, mismanagement of funds and resources by the local governments and authorities have been recognized as another factor contributing to poor ANC services.

The experts highlighted how health systems in their settings show gaps between the recommended practices and the care that patients actually receive. These gaps and related inconsistencies make it difficult to collect accurate data at all levels, thus diminishing the opportunity for programmatic interventions to benefit the population.

##### Health workers’ attitudes and shortage of staff

In many countries the shortage of skilled healthcare workers, and consequently a tired, overworked and fatigued workforce, is contributing to poor ANC. Moreover, coupled with lack of maternal care training, what some experts have experienced on the field is the disrespectful care towards expectant mothers and abuse that some women have to endure while seeking care.

#### Community level

##### Stigma and empowerment, gender-based disparities

The emergence of stigma and gender-based disparities at the individual level are closely related with the community. Many women experience some form of mistreatment, as abuse, neglect and discrimination when seeking care.

As discussed before, women’s ANC seeking behaviour is restricted by the significant role of their family members in health care decisions. Communities and community leaders play a significant role in influencing and affecting women access to maternal health services, especially in rural communities. Gender norms, values and expectations about how women and men should be and behave are usually shaped by the community. Although these norms are specific to particular cultures and societies, there is strict gender role distribution when it comes to the issue of taking care of deliveries and childbirth.

Health-related stigma is a complicated phenomenon rooted in social inequity and power imbalance. Discrimination, oppression and marginalization, as enacted and reinforced by the community, frequently have negative social, psychological, behavioural and medical consequences for people needing care. Stigma and discrimination at community level and the consequent fear of judgment, affect the willingness of women to access ANC services.

##### Urban vs rural differences

Many of the experts have highlighted the urban vs rural differences in the determinants of maternal health service utilization. They have suggested that the inadequate use of ANC services is more of a rural phenomenon, which is linked to individual level challenges discussed above, as woman’s education level and autonomy. Inadequate ANC was mostly prevalent among poor and low empowered women who reside in rural areas.

#### Policy level

##### Midwife-led and preventive care

Many experts have acknowledged and recognized the role that healthcare providers, such as midwifery maternity care, can have in addressing psychosocial risk factors, which are often not given enough recognition. In fact, inadequate psychosocial assessment and or support can have adverse effects on both the mother and the foetus. Maternity care involving midwifes as the primary care provider leads to positive health outcomes for both mothers and their babies. In addition to improving maternal and neonatal health, better integration of midwifes into the health systems across regions can facilitate the reduction of primary care shortages across such areas.

The concept of preventive care in the form of early risk identification was also discussed, highlighting the importance of collaboration and communication between healthcare professionals and patients in the provision of comprehensive and holistic care.

##### Regulatory challenges, decentralization

The effectiveness of ANC services depends on the availability of a quality antenatal model that is implemented by the local government. Experts highlighted how poor regulatory mechanisms, or insufficient capacity to enforce regulations, contribute to the difficulty in assuring quality of care in public and private ANC clinics.

### ANC access and delivery: solutions to improve it

Based on the initial presentations and experts’ experiences, participants were asked to think of an innovative approach to improve ANC access and delivery at different levels. The conceptual framework for MNH innovation based on the WHO’s health system and the building blocks and the Tanahashi model of measuring health systems performance (Fig. 2) was used to present the discussion points along the continuum of care to expectant mothers.

#### Health service delivery

The experts have stated that innovative approaches in ANC should aim at improving the quality of health service delivery along the continuum of care. Delivering and ensuring access, quality and safety of care across different locations and over time is deemed fundamental. Experts agreed that more attention should be drawn at implementing a care model that requires an in depth understanding of the user’s perspective, which should be inclusive and aiming at reducing the challenging barriers to care. Additionally, it was acknowledged that mental health care needs to be integrated into all elements of health, particularly in ANC, where evidence suggests that increasing resources for mental health care is linked to better health outcomes for both mothers and their babies. Thus, the emphasis was put on the development of counselling packages and community education on maternal and neonatal health, which should better integrate the health needs of the population and available resources, as healthcare providers, medicines and money.

#### Medical products and health technologies

Equitable access to medical products and health technologies of assured quality, safety and efficacy is fundamental for a functioning health system. Strategies to make novel medical products and health technologies available in LIMICs are not implemented enough. Barriers to the efficient implementation of new technologies have been described by the experts as a governmental and stakeholder problem, which should establish international norms and standards to promote the quality of medical products. More support should be offered, through guidelines and strategies that can maximize patient and staff safety and that can promote equitable access to it. Many experts have acknowledged that the reliable collection of data in LMICs is challenging due to multiple factors, such as human resource, capital and technological factors. More investments should be made to improve data collection and consequently to increase the utilization digital health and digital health tools, which have the potential to meet the challenge of shortage in human resources, and to reach poor, rural and undeserved areas. Therefore, the experts have highlighted that the integration and incorporation of health technologies in the primary care system, has the potential to ensure universal access to maternal-health related information e.g., by delivering financial incentives through mobile money technologies and enabling remote access of maternal health services. This could allow more individuals, living in rural, remote and marginalized communities, to access services which remain under-utilized, such as ANC.

#### Health workforce

As discussed above, the healthcare workforce is often tired and overworked and staff shortages are becoming a reality in many rural settings across LMICs. Countries and stakeholders should harness more innovative workforce approaches, involving novel and/or additional training programs to improve the supply side of maternal care. Countries should finance the scaling-up of education programs for healthcare staff in a realistic and sustainable manner across settings.

#### Health financing

Addressing financial constraints and the financial barriers that limit the access to quality care has been highlighted by the experts across settings as an important concern across health system. The creation of innovative financial programs that aim at attenuating financial barriers to ANC and at improving the coverage of such care should be central in government’s policy plans. The concept of “value for money” has also been touched upon, becoming a central topic in the development of a comprehensive ANC policy and its delivery. The first level in generating value is to secure the value of health policies, especially to measure the value for money in the choice of policies to fund. In this context, the value of money refers to using economic methods (usually cost-effectiveness analysis) to measure the health promotion achieved at a particular level of spending.

Moreover, a multidisciplinary approach to health financing has been also discussed. In fact, the experts have expressed a positive view on the promotion of international dialogue to increase ANC financing across settings, from domestic and external sources, and to ensure that the new sources contribute to the development of sustainable national financial institutions.

#### Community ownership and participation

Innovative approaches are increasingly aimed at strengthening community health mechanisms that improve links with primary health care. Across settings, it was recognized that community-based interventions, facilitated by community health workers (CHW), and women’s groups, can be a really useful tool in strengthening ANC. Women’s groups aim to empower and support their members; engaging in conversation with peers helps normalize the experience of pregnancy and share knowledge in the community. The group format also promotes self-efficacy and social support for pregnant women by creating a forum for participants to develop skills and confidence, share experiences and resources.

The empowerment of women and CHWs, and the role of the group consultation model, was highlighted across experts as a fundamental and innovative step aimed at reducing the logistical and economic burden of ANC attendance. In fact, group ANC models, which aim to put women at the centre of service provision in order to improve women’s access, engagement and satisfaction with care, can increase convenience for women and providers and can make care delivery more efficient. For example, as long wait-times for care have been mentioned to hinder MNH services, scheduling group sessions in advance could help to reduce this challenge. This solution, in addition to allowing all women to receive the recommended care, it allows women to access counselling opportunities, which benefits both women and the healthcare providers. Such collaboration between providers and women enables both efficient and comprehensive care delivery, which may improve care provision, experience, and utilisation while also providing opportunities to ensure continuity of care [10].

#### Leadership and governance

Innovative leadership and governance initiatives related to the formation of partnerships and the establishment and implementation of national ANC policies have been discussed. Across sectors of government, and with actors outside government, including civil society, innovative approaches to generate support for policy and influence key determinants of health, should be considered. Partnerships for ANC include public-private cooperation between government and regional or international associations, to enhance capacity and quality of ANC delivery. In order to do so, leadership should facilitate the collection of quality-assured, timely routine data, managed by innovative new interventions that would improve and strengthen the accuracy of routine real-time health data, contributing evidence for ANC policy-making.

## Discussion

### Challenges

Across regions, experts emphasized that many barriers and norms, largely associated with community cultural beliefs, and influenced by the traditional household set-up, limit expectant mothers’ autonomy to seek care. In fact, in many instances, women are expected to prioritise the health of their family over their own, and to continue to work both outside and inside the home [11]. Furthermore, socially constructed gender norms continue to hinder men’s participation in pregnancy and childbirth. Men control decision-making at home, which influences the timing of ANC attendance [12]. Similarly, men control the economic resources of the household, which in turn influences women’s choice and ability to use maternal and child health services. Men and the community still view pregnancy as a typically feminine domain and do not feel involved. Pregnancy is still a stressful event that disrupts the link between families and communities. Women are often burdened by physical, psychological, and financial hardships, coupled with inadequate care of ANC, skilled birth attendance coverage and transport facilities. These results are consistent with previous studies [13].

Consistent with the literature, many experts have emphasised that women’s beliefs and attitudes play a role in deciding whether to initiate or continue ANC. There are numerous shared cultural experiences when the patient seeks care; their cultural background, the culture of their provider, and the medical culture. Patient have different beliefs, attitudes, values and behaviours to healthcare providers. The culture of the medical community is often at odds with the patient’s culture; i.e., education, means of transport, occupation and socio-demographic factors, and when they fail to recognize these differences, they may deliver low-quality care. Such differences can also influence women’s decision making, acceptance of care and can make it difficult for patients to follow advice from the medical community [14]. Thus, especially in pregnancy care, a doctor should have a basic understanding of patients’ needs and communicate with them effectively in a way that makes them feel comfortable. Doctor’s interventions must consider broader economic, geographical and social factors that might affect an individual’s access to services. Finally, it is essential to have cohesiveness between culturally-appropriate services and other health care providers that women and their families encounter along the continuum of care through pregnancy [15].

Additionally, another significant aspect highlighted by the experts in respect to the challenges of ANC delivery, was the reported limited understanding that women have on the purpose of early ANC. This lack of understanding was suggested to associate with women’s educational level and literacy rate. In fact, it has been shown that the increase in women’s educational level is a major motivator for increasing the likelihood of her ANC attendance [16, 17]. This suggests that educated women are more likely to have adequate knowledge of prenatal care services and understand the importance of early booking for ANC as well as attending the recommended eight visits [18]. Thus, they tend to value ANC and will use pregnancy care services more, compared to less educated women. Longer time in school can also develop women’s ability to reach out to health workers to ask questions and discuss possible health issues [19].

Programs to promote health education among expectant mothers with low levels of education are necessary to raise awareness among rural women about the benefits of optimal ANC. In order to do this, it is important to strengthen the existing role of CHWs, who are able to provide appropriate health education and create connections between vulnerable populations and healthcare providers [20]. Thus, it is imperative that significant efforts be made to improve the quality of ANC by providing pregnant women with appropriate counselling, including supportive listening, advice, and relevant information. Involving mothers as active participants in the decision-making about their care would also help make changes in health care, from one based on provider-dominated dialogue to one that involves clients in the decision-making process. This requires a transition in the role of health workers from one of authority to one that is based on collaboration and partnership between patients and providers. Pregnancy information should also be provided in a form that is easy to understand and accessible to users of ANC services. Healthcare providers’ statements on reproductive health issues need to be adapted to different social contexts, including those with low levels of education and income. Furthermore, there is a need to continue to focus on community education and awareness campaigns on the importance of early participation in ANC. Educational interventions targeting both men and women have been reported to improve the health-oriented behaviour of pregnant women and improve birth preparedness and complication readiness. In addition to home visits, education could be offered at various political and social gatherings in the community. In Tanzania, community health workers are reported to play an important role in promoting men’s participation in maternal and child health issues [21]. A key question for practitioners and policy makers is how to improve women’s perception of the importance of pregnancy care. Any intervention should be culturally relevant. Training programs in cultural literacy and sensitivity should be developed to improve healthcare utilization among women. Stakeholders may want to include cultural skills and sensitivity training in its health education curriculum when training new health professionals [22].

Finally, a recurrent theme in the expert consultation was namely that geographical inaccessibility contributes to the late onset of ANC attendance. This finding has also been reported in other studies. In fact, Nsibu and colleagues showed statistically significant associations between the place of residence and attendance to the first prenatal visit in the first trimester [23]. Additionally, a great discrimination in the allocation of resources and in the availability of rural and urban health facilities was described. Poor reediness to provide antenatal/natal health services and supplies is hindering the ANC landscape. In fact, differences have been documented in availability of equipment and supplies needed for antenatal and natal services between urban facilities and rural ones [24].

It is important to ensure greater use of ANC services, by establishing health care facilities in catchment areas in rural communities, employing more qualified health workers to provide medical care to women in their communities, and ensuring adequate transportation infrastructures and services. Stakeholders need to ensure that maternity care services are closer to home, which could be achieved, especially in rural and hard-to-reach areas, through mobile clinics that would help many women with financial difficulties access prenatal care.

### Solutions

Reshaping the health system requires political leadership and policy change, hospitals that can provide high quality, respectful maternal and childcare, health systems that can break down barriers to access, and empowered populations that can demand high quality care [25, 26]. Improving nurses’ and midwifes’ knowledge of comprehensive care and its contribution to the quality of care is an important issue that needs to be addressed in many countries where existing nursing practices are unsatisfactory and the doctor-led care is commonly applied in clinical environments. The central role of nurses and midwives in delivering respectful, caring, friendly and helpful ANC can be seen in many studies throughout the literature. For example, many studies have showed that the presence of providers who were caring and sympathetic, and familiar with patient’s cultural practices and communities, were essential factors in encouraging ANC demand and usage [27, 28]. Through the identification of the needs of patients who are neglected by sole use of the doctor-led care recovery can accelerate, hospital stays can shorten and the costs reduced [29].

As discussed earlier, barriers to access maternal health services using telehealth relate to common challenges, as limited access to broadband in rural areas, cost of the equipment, scheduling time with providers [30]. Due to the scarcity of health resources, particularly in developing countries where those are often very limited, only appropriate (effective, safe and feasible) technologies should be implemented and used. There are different opportunities to use telemedicine to expand access to maternal health care for women living in rural areas; however, its acceptance remains limited. Based on the current literature, technological anxiety and perceived risk act as significant barriers to telemedicine usage. Kamal and colleagues [31] have demonstrated that technological anxiety had a significant negative relationship with telemedicine usage intention. In fact, because people living in developing countries don’t frequently access medical care, they prefer face-to-face meeting with doctors, instead of remote mode of communication. Additionally, they have noted that, due to already inadequate and poor resource, people associate a perceived sense of risk with the adoption of telemedicine [31].

Nonetheless, digital health technologies overall hold promises for addressing major public health backlogs and for strengthening health systems in LMICs. New technologies have the potential to harness clinical and public health, and more research is needed around emerging ones, including artificial intelligence, big data, cloud, cybersecurity, telemedicine and wearable devices to demonstrate their potential use in remote settings. For example, a recent case reported by Runckle et al. [32], have showed that the use of wearable sensor technology in prenatal care, was well received by both patients and providers, which responded favourably to the implementation of such technology, especially in rural underserved populations.

From the need of new technologies, to address the healthcare staff shortages occurring in resource-limited areas, addressing financial barriers that many countries encounter is fundamental. Thus, it is important to ensure value for money through an integrated people-centred health service approach. This approach should consciously incorporate the perspectives of individuals, families and communities and should see them as participants and beneficiaries of a trustworthy health systems that address their needs and preferences in more holistic way [33]. Different examples of people-centred and integrated health services can be found in literature. In Mali, primary care networks have been developed, which are made up of community-owned, community-operated primary care centres with the support of government-run district health teams. In rural South Africa, nurse-led chronic disease management programmes focusing on people with high blood pressure, diabetes, asthma have supported patient education, self-management support and improved surveillance leading to improved control of disease. In South Sudan, Uganda and Zambia there have been cases of integrated community case for the management for malaria, pneumonia and diarrhoea to reduce child mortality, involving community health workers who assess and treat children with serious illnesses [34].

Ensuring equal access to quality health services that meet the broad needs of individuals and communities requires a fundamental change in the way health services are planned, funded and delivered. Thus, it is essential to engage and empower individuals, families and communities so that they have the opportunity, skills and resources to develop into articulate and empowered users of health services. Hence, policy interventions should actively promote health literacy, shared decision-making and patient self-management contribute to the health services that people value most. For instance, a study investigating the association between access to health care and women’s empowerment in Myanmar, have found that, especially in rural areas, women’s empowerment was an important factor of one’s ability to access care [35].

## Conclusion

The expert consultation offered the opportunity to share experiences around challenges of maternal and newborn care from different settings, and brainstorm on possible approaches to innovative solution. Overall, the event strengthened the idea that the burden of poor maternal health, morbidity and mortality is concentrated among vulnerable populations. The findings suggest several courses of action for improving ANC services and delivery in LMICs and rural areas. Good quality, equitable, evidence-based maternal health services that respond to local needs and are capable of meeting emerging challenges should be prioritized. Investments in strengthening health systems, including data and surveillance systems, facility capability, and a skilled health workforce should be considered a fundamental step towards better maternal health services.

This paper contributes to the idea that ANC services and delivery in LMICs and in resource-limited settings continue being disrupted by different challenges and barriers. The COVID-19 pandemic had further highlighted these obstacles [36]. Innovative measures are required to address these obstacles and ensure women are not denied access to available, accessible, acceptable, and high-quality maternal healthcare services in normal situations and in spite of emergencies, such as COVID-19.

Efforts to train and motivate healthcare providers to adopt online, remote approaches such as use of telemedicine are critical. Similarly, initiatives to expand the involvement of frontline maternal healthcare providers and to deliver information on the availability of services strategies are crucial to mobilise and secure community confidence in the safety of maternal care services.

This expert consultation emphasized ANC delivery – especially in resource-constrained setting – as a wicked problem [37]. The experts’ views highlighted how the ANC challenge takes a different form, depending on the vantage point and domain of observation: in some contexts, it is an issue of shortage of healthcare infrastructures, in others gender bias, domestic violence and power dynamics are predominant antecedents, in yet other domains women education and the characteristics of the built environment are perceived as the predominant foci of attention. More holistically, and in line with the socio-ecological view, complex interactions among all these elements are foundational to understand and properly address ANC delivery in different settings. Solutions also need to be multifaceted, and mindful that one intervention might lead to unexpected adverse consequences, according to the wicked problem framework [38, 39]. For example, whilst digital technologies hold enormous potential for improving ANC services in remotes, without adequate financial support and policy intervention there is high risk of increasing income-based inequity in ANC services and outcomes.

## Data Availability

The authors declare that the data supporting the findings of this study are available within the article and its supplementary information files.

## Abbreviations

ANC: Antenatal care
MMR: Maternal mortality ratio
SDG: Sustainable development goals
LMIC: Low-middle income country
HIC: High income country
SEM: Social ecological model
MNH: Maternal and newborn health
GP: General practitioner
CHW: Community health worker
